# Beyond mobile populations: a critical review of the literature on malaria and population mobility and suggestions for future directions

**DOI:** 10.1186/1475-2875-13-307

**Published:** 2014-08-09

**Authors:** Catherine Smith, Maxine Whittaker

**Affiliations:** School of Population Health, Herston Campus, University of Queensland, Brisbane, Qld 4006 Australia

**Keywords:** Mobile populations, Imported malaria, Border malaria, Community engagement, Malaria elimination

## Abstract

**Background:**

Although population mobility is frequently cited as a barrier to malaria elimination, a comparatively small body of literature has attempted to systematically examine this issue. This article reviews the literature on malaria and mobile populations in order to critically examine the ways that malaria elimination experts perceive the risks surrounding population mobility. The article brings in perspectives from HIV/AIDS and other infectious disease control programmes working in areas of high population mobility. The article aims to move beyond the current tendency to identify mobile populations as a risk group and suggests ways to reconceptualize and respond to population mobility within malaria elimination.

**Methods:**

The review was commissioned by the Asia Pacific Malaria Elimination Network (APMEN). Searches were made using PubMed, ProQuest, Google and Google Scholar. The review includes English language published peer-reviewed literature and grey literature published up to November 2013.

**Results:**

The review identified three key themes in the literature: mobility, economic development and shifting land use; concerns about accessing mobile populations; and imported and border malaria. The review found that the literature treats mobile populations as a homogenous entity and is yet to develop a more accurate understanding of the true risks surrounding population mobility. Concerns about accessing mobile populations are overstated, and methods are suggested for working collaboratively with mobile populations. Finally, the review found that many concerns about mobile populations and imported malaria would be more productively framed as border health issues.

**Conclusion:**

The focus on mobile populations is both excessive and insufficiently examined within the current literature. By its very nature, population mobility requires malaria elimination programmes to look beyond isolated localities and demographic groups to respond to the interconnections that mobility creates between localities and population groups. Malaria programmes will gain greater clarity by refocusing the discussion away from mobile populations as a risk group and toward mobility as a system involving interconnected localities and multiple demographic groups. Rather than focusing on mobile populations as a risk group and a barrier to elimination, malaria elimination programmes ought to develop collaborative community engagement efforts in border areas and regions of high population mobility and where imported malaria is of concern.

**Electronic supplementary material:**

The online version of this article (doi:10.1186/1475-2875-13-307) contains supplementary material, which is available to authorized users.

## Background

Much concern has been expressed over the past decade about the impact that population mobility bears on malaria transmission. Many have claimed that globalization and increased border porosity has had a significant impact on the nature of global health generally [1–4], while a substantial literature has described human population movement as a major challenge facing malaria elimination [5–10]. However while the malaria elimination literature often describes mobile populations as a risk factor and a barrier to elimination, a comparatively small body of work has attempted to systematically research this issue or look for solutions. This is, perhaps, because compared to many other infectious disease control programmes malaria research only rarely employs technical expertise in the social sciences [11, 12]. While it is generally accepted that programme strategies ought to be evidence-based, this lack of engagement with social scientific research has disempowered malaria control programmes and researchers from clearly identifying the significance that population mobility has on malaria transmission and from developing strategies to address these concerns.

This article seeks to identify the risks that malaria scientists and programme managers assume to be associated with population mobility and to make recommendations to support malaria elimination programmes to address the challenges of population mobility. The review found three major themes within the literature on malaria and population mobility. These are: i) economic development and shifting land use; ii) accessing mobile populations; and iii) imported malaria, especially importation in porous border regions. The article discusses the central points raised in each of these three themes, before bringing in some key lessons learned by HIV/AIDS programmes working with mobile populations. The article critically analyses the literature with the intention of supporting malaria elimination programmes and researchers to move beyond describing mobile populations as a risk factor and to find constructive solutions to the challenges assumed to be associated with mobile populations.

## Methods

The article was commissioned by the Asia Pacific Malaria Elimination Network (APMEN) and an early draft of this article was presented at the Annual Technical and Business Meeting of APMEN in Seoul, Republic of Korea in May 2012. This review includes published, peer-reviewed English language literature sourced through PubMed and ProQuest and grey literature sourced though Google and Google Scholar. The authors used the search terms: malaria and mobile populations; malaria and mobility; border malaria, and; imported malaria; and included articles published until November 2013.

The authors excluded a large number of articles that contained references to malaria and population mobility, but which were in fact concerned primarily with other issues and which did not attempt to engage in a substantial discussion of population mobility. The author prioritised articles that attempted to discuss the relationship between malaria transmission and population mobility in some detail and case studies of attempts to address population mobility, which left 70 articles specific to malaria and population mobility. The author used open coding to identify the primary themes discussed in the article, and the secondary assumptions that underpinned the article. This highlighted the three key themes of rural economic development and shifting land use; concerns about accessing mobile populations; and imported and border malaria, together with a number of secondary assumptions about mobile populations that the author identified as recurring across the broader literature. The article is structured around these three key themes and the assumptions underpinning them.

Since the intention was to find innovative ways to address population mobility, the authors then searched the same databases for lessons learned from other infectious disease control programmes, especially HIV/AIDS, polio and smallpox control programmes. HIV/AIDS literature was quickly determined to bear the most relevance for the purpose of this review, since there is a well-developed literature on HIV/AIDS and population mobility that is based on strong interdisciplinary scholarship. Given the large body of literature on HIV/AIDS, the authors prioritized applied research and policy documents directly aimed at helping policy makers to strategize an approach to population mobility. No preference was given to geographic focus of the research or to the publications of APMEN partners.

This literature review is based on a reading of 102 articles. Of these 70 related specifically to malaria and population mobility; 12 offered policy recommendations on HIV/AIDS and population mobility; 4 focused on polio and population mobility; 1 article discussed human population mobility and dengue fever with reference to malaria; and 11 were general commentaries on population mobility and public health that were returned in a general search on population mobility and malaria. This article cites the articles that offered the most productive discussion of the themes that recurred throughout the literature. A full bibliography of the articles informing this literature review can be found in Additional file 1.

The authors acknowledge that there is a likely gap between research and practice, and that this is a possible limitation of this article. Although the review includes some grey literature that is available online, discussions at APMEN meetings indicate that it is likely that countries have developed strategies for working with mobile populations that have not been published and are therefore not able to be included in the review. As such this review should be read for its intended purpose, which is to critically review the assumptions surrounding population mobility within the English language malaria literature, and to make recommendations to find more productive ways to describe and strategize these concerns. This possible gap also illustrates the value of documenting country experience in order to develop better strategies for addressing population mobility. This article was not subject to review by an ethics review committee as it is based on a desk study alone and did not involve any research with animal or human subjects.

## Results and Discussion

The review found three sub-bodies of literature within the broader discussion on malaria and population mobility. These are: economic development, shifting land use and population mobility; concerns with accessing mobile populations; and imported or border malaria.

### Economic development, shifting land use and population mobility

One of the longest running and most well developed themes within the current literature looks at the relationship between economic change, land use and population mobility. Beginning from the early 1990s malaria researchers began to examine the ways in which rural economic development generated population movements that affected malaria ecology. Although rural economic development is seen to lead to a reduction of malaria in the long through reducing vector-human contact, rural development projects can also lead to increases in malaria or shifts in the spatial distribution of malaria as new industries and townships develop in areas that remain endemic despite economic growth [13–16].

Such population movements present a challenge to malaria control programmes since they require an understanding of how the spatial distribution of malaria shifts through time and across multiple locations that become interconnected through population movements. This draws attention to the spatial and temporal dimensions of mobility, as well as to the demographic makeup of population movements. The development of new rural economies such as plantations, rubber tapping industries, forestry work, the construction of dams and rural infrastructure may bring non-immune people into endemic areas [17, 18]. Likewise the cessation of rural economies may drive local populations into other rural areas that may be endemic [15, 19, 20]. Highways that pass through endemic areas allow people and malaria parasites to move between localities that may otherwise carry different malaria risks [13, 21].

An illustrative example can be found in Barbieri and colleagues’ work in the Matto Grosso in Brazil [13]. Rather than mapping malaria risk on the basis of microlocality or demographic group alone, their research begins with the assumption that malaria risk will shift continually as malaria ecology changes and as economic processes drive populations into, out of and through endemic areas. While the Matto Grosso as a whole has a high prevalence of malaria, the authors observe that infection is unequally distributed within the region. The distribution of malaria within the region has been influenced by deforestation, the development of highways and the expansion of the agricultural sector, all of which have shaped both the ecology and the demographic makeup of the region and influenced the risk activities that affect exposure to vectors. Soy plantations, cattle ranches and smaller farms began to be established in the Matto Grosso beginning in the 1970s and expanded rapidly with intensifying deforestation of the Amazon Basin in the 1990s. As large cattle ranches become more common cattle herders are becoming increasingly sedentarized. Those who remain mobile are more susceptible to infection and more likely to transmit parasites to workers at cattle ranches and those living in the new urban settlements in the same district. As the Matto Grosso continues to expand into previously forested areas, malaria prevalence continues to fluctuate and to be shaped by the changing agricultural economy, the establishment of new urban settlements and the construction of highways, all of which influence the patterns of population movement and malaria transmission through the Matto Grosso. The authors draw from cross-sectoral expertise in their study design and worked with agricultural planners to integrate health risk assessments into long term agricultural planning.

Like Barbieri and colleagues, many point out that changes in land use and rural economic development is often the direct result of government or development policy, and as such can be reasonably foreseen and responded to with cross-sectoral collaboration by governments, aid agencies and other relevant stakeholders [13, 15, 16, 20, 22–24]. In the GMS, for instance, much academic research and grey literature has analysed regional trends in population mobility, enabling malaria researchers to analyse and apply this knowledge to shifting malaria transmission in the region [15, 21]. The Cambodian government has developed a strategy for engaging mobile populations through ensuring access to prevention measures, early diagnosis and treatment, behavioural change communication, research and better surveillance and management [25].

While shifts in malaria risk associated with rural economic development can often be forecast, some endemic areas become populated faster than the expansion of health care services and malaria control activities. As Jitthai points out for the Greater Mekong Subregion (GMS), while the farms and plantations that attract migrant workers are located in rural areas they are not remote but to the contrary are well connected to roads and transport networks, including the Asian Highway that cuts across the GMS [21]. In other words while accessing malaria endemic rural areas may present operational challenges for malaria control programmes, these endemic areas are no longer isolated but often localities that are becoming highly interconnected. Continuing to see these locations as remote is counterproductive since these points of interconnection are potential points of entry for malaria control programmes to carry out prevention and treatment programmes. In this sense the challenge for malaria control is not the rapid pace of population movement but the need for malaria experts to stay informed about and responsive to socio-economic changes affecting malaria transmission in a particular region, including through interdisciplinary and cross-sectoral engagement [12].

As demonstrated in Brazil [13] and the GMS [21], one reason that population mobility presents a challenge to malaria control is that it connects localities that otherwise carry heterogeneous malaria risks. As population mobility becomes better integrated into malaria control strategies it will be important for malaria control programmes to better understand the ways that population movements link otherwise disconnected localities. As explained in detail below, malaria control programmes will be better equipped to stay responsive to these changes if they shift away from a focus on mobile populations as a demographic group and towards a focus on mobility as a system involving multiple demographic groups extending across localities.

### Accessing mobile populations

One of the prominent concerns within the recent literature focused on malaria elimination is the assumption that mobile populations are hard-to-reach. Much of this concern with access stems from an imagining of mobile populations as completely segregated from local communities, moving rapidly across national borders and as engaged in illegal activities that make them likely to avoid health authorities. The preliminary research that has attempted to work with mobile populations indicates that many of these assumptions are overstated as are the difficulties in accessing mobile populations. By adapting lessons learned from HIV/AIDS programmes, the authors recommend that malaria control programmes will be better able to access hard-to-reach populations when they: (i) more accurately identify the malaria risk associated with various population movements; (ii) shift from a focus on mobile populations as a risk group to mobility as a system involving multiple demographic groups and extending across geographical locations; and (iii) use collaborative and participatory methods that work with communities rather than targeted campaigns that describe mobile populations as a risk factor.

#### i. Understanding mobility and malaria risk

It is striking that the commonly used concept ‘mobile populations’ suggests that the term refers to a salient demographic category in itself. In reality, the term mobile populations incorporates a wide variety of demographic groups, engaged in very different kinds of mobility, who are integrated into their communities in markedly different ways and who carry varying malaria risks. Mobile populations commonly associated with malaria include forestry workers [19, 25, 26]; agricultural labourers [13, 16, 20, 21, 27]; fisherfolk [20]; and daily crossers living in porous border zones [19, 23, 27–34]. They may also include construction workers [18, 35], traders [35]; transport workers [15]; displaced persons [18, 23, 29, 36–38], indigenous groups [39] and international or domestic tourists, which increasingly includes tourists from a second endemic country [40]. Mobile populations also include soldiers; people returning to help families at harvest time or population movements associated with national holidays, pilgrimage or other religious or cultural practices that involve large scale population movements [9, 12]. Urban populations are rarely discussed in the literature but may also be affected by large-scale population movements through endemic areas [41]. In short, population movements are much more heterogeneous than the term ‘mobile populations’ suggests, and abroad array of demographic groups participate in mobility systems that shape malaria transmission [8, 11, 17, 21].

As noted by Pindolia and colleagues, the malaria risks associated with diverse mobility practices differ significantly and insufficient attention has been given to identifying the factors that increase and mitigate the risk of malaria transmission in particular contexts [8]. For example, little attention has been given to assessing the ways that the timing and duration of mobility shapes malaria risk, and some preliminary research suggests that the speed of mobility is not always as significant as previously imagined. Tatem and colleagues, for instance, examined the extent to which frequent travel between Zanzibar and mainland Tanzania presented a challenge to elimination efforts [42]. The researchers examined the risk that this mobility posed to malaria transmission by using anonymized data from the phone call records to measure the scale and duration of mobility from Zanzibar to mainland Tanzania and to determine the number of people moving to high risk areas [42]. Contrary to expectation, the majority of human traffic was to low risk areas and most people returned within five days, posing little significance to malaria transmission. The actual patterns of mobility were in fact quite different from the picture assumed by malaria researchers, and in fact movement to the mainland poses only a minor challenge to Zanzibar as it heads towards elimination.

Likewise the duration of mobility does not always prevent access to treatment. For instance researchers on the Thai-Cambodia border found that while their project was located in a region of high mobility, that mobile people stay within a region long enough for follow up medical care, in particular for the duration of a 28-day drug treatment [43]. The same study found that some health clinics didn’t offer treatment to mobile people under the false assumption that they would not be in the location long enough for a full course of treatment. This shows that unfounded assumptions about the rapid speed of mobility not only present a lost opportunity to develop more responsive programmes, but can also contribute to the risk of cross-border transmission by denying services to mobile populations [43].

Similarly a project in the border regions in the Yunnan province of China also found that while local communities were highly mobile and frequently crossed into Myanmar and other neighbouring countries to marry, meet family members and carry out economic activities, that most of the study participants had lived in the study area for more than ten years and were well integrated into communities [34]. The authors concluded that the significance of population mobility has been overstated, and that poor access to health services was a more significant risk factor than cross-border mobility.

A small number of researchers have begun work towards more accurately identifying the risks associated with various kinds of population movements. Building on Stoddard and colleagues’ model for population movement and dengue fever, Pindolia and colleagues have begun to develop a model to quantify the risks of population movement on the cross-border transmission of *Plasmodium falciparum* [8, 44]. They point out the need to prioritize population movements with the most significance to malaria and to identify the factors that mitigate these risks without the need for direct intervention from malaria elimination programmes [8]. Similarly, Wesolowski and colleagues combine quantitative data on large scale population movements sourced from mobile phone data with mapping of malaria prevalence to identify the human population movements most significant to malaria transmission and to locate significant hotspots in Kenya [45].

Working in the GMS, Canavati and colleagues propose three semi-quantitative indices that can be used to identify mobile populations most at risk of malaria [25]. First, a forest/malaria exposure index takes into account factors such as proximity to forest, duration of stay in forest, housing conditions, and use of ITNs and other preventative measures. An access/outreach index takes into account the point of human-vector contact, the work and living locations of the risk group, access to prevention and control activities and access to diagnosis and treatment. While a malaria vulnerability index takes into account knowledge of malaria; immunity status; ITN ownership and use; and housing conditions. The indices are designed to enable programme managers to compare the malaria vulnerability of particular groups and target their programmes accordingly. Finally Bloland and Williams identify the factors that increase malaria risk during complex emergencies characterized by the rapid, large-scale and forced displacement of populations [36]. In addition to the risk factors identified above, overcrowding, the existing overall health of the displaced persons and concurrent pressures placed on infrastructure in the region, are among many factors that shape malaria risk in complex emergencies.

As noted by Pindolia *et al.* [8], Prothero [12] and others, data on population movements and the expertise to analyse it already exists and can be accessed through cross-sectoral and interdisciplinary engagement. For example, a 2013 supplementary edition of the Southeast Asian Journal of Tropical Medicine and Public Health on malaria and population mobility in the GMS offered a number of articles addressing malaria in the region from various disciplinary perspectives [11]. This special edition stands as an example of one format through which interdisciplinary dialogue can be generated in a manageable format that helps to inform a response to population mobility.

#### ii. From mobile populations to mobility systems

Another prominent concern within recent literature is the identification of isolated high risk populations, or hotpops (hot populations). While it is important to understand the epidemiology of malaria in varying contexts, the experience of HIV/AIDS programmes illustrates a number of reasons why it can be counterproductive for public health interventions to focus on risk populations. Instead of focusing on mobile populations as a high risk group, many HIV/AIDS programmes approach mobility as a social process that is driven by a range of social, economic and cultural factors, that involves multiple demographic groups and that extends across geographical localities.

Firstly, approaching mobility as a system would better equip malaria elimination programmes to access populations through social networks, peer educators, participatory development and other forms of community engagement. Recognizing that mobile populations are part of the same communities as their more sedentary counterparts, many HIV/AIDS programmes now advocate a focus on high-risk situations rather than on high-risk groups [46–49]. Skeldon argues that HIV/AIDS programmes need to focus on “all points of a mobility system” rather than a singular focus on mobile populations [48]. In the case of HIV/AIDS prevention programmes, Skeldon suggests involving people such as border guards, employers of contract labourers and those working in industries indirectly connected with commercial sex work such as taxi drivers and bar workers. The purpose here is not only to access hard to reach people, but to work effectively with broad groups of people at risk of HIV/AIDS.

Secondly, recognizing mobility as a system will allow malaria elimination programmes to identify how localities become interconnected through human population movements and how the spatial distribution of malaria changes through time. Within the malaria literature, this is best illustrated through the literature on rural economic development and shifting land use reviewed in the first section of this article [13, 19–21], and through work that focuses on the spatial dimensions of population mobility [8, 10, 42, 44, 45]. HIV/AIDS programmes offer insight on how interventions can be designed based on this knowledge. For example, the Asian Development Bank and other HIV/AIDS programs in the Greater Mekong Subregion recommend implementing interventions at both origin and at destination towns for migrant workers, which are often on both sides of a national border [46–49]. They also implement interventions along the path of movement, such as along highways and trade routes. Finally they target points of congregation where many groups come together such as border towns, market towns and popular rest places. In the case of cross-border malaria transmission this may involve establishing programmes on both sides of a border, in townships in endemic areas, in towns along highways that connect endemic and receptive areas, and other potential sites of transmission.

Finally, to the extent that mobile populations are hard-to-reach, this approach to mobility is more likely to avoid stigmatizing targeted groups and allow for more successful engagement with populations at risk and the broader community. Many large HIV/AIDS programmes recognize that top-down approaches can be counterproductive when working with marginalized people, to the point that they can undermine the efficacy of programmes [47–49]. HIV/AIDS programmes illustrate the value of working collaboratively with communities, especially if they are wary of authorities. Many of the most successful HIV programmes emerged from communities themselves and were supported by government agencies [47]. When top-down approaches have been implemented with hard-to-reach groups, these are more successful when they target the whole community first before moving on to a specific group, who may feel stigmatized or vulnerable [47]. As Williams and colleagues point out, this strong focus on mobile populations as potential transmitters of malaria also fails to recognize that mobile populations themselves are a vulnerable group at risk of malaria, who themselves share a stake in malaria control and elimination [37], see also [47]. Brentlinger illustrates that the global distribution of malaria has been shaped by a long history of human rights abuses and socio-economic inequalities that have led certain groups to become structurally vulnerable to malaria [50]. Hence there are ethical as well as pragmatic advantages of shifting away from a focus on mobile populations as ongoing sites of infection, and towards approaching mobility as a multi-faceted system.

#### iii. Methods for working with mobile populations

Focusing on mobility as a system involving multiple demographic groups also opens up opportunities for a better engagement with mobile populations through the use of social networks, peer educators, participatory development and other forms of community engagement. Many of the assumed difficulties in access mobile populations stem from the assumption that mobile populations are completely segregated from the broader community. Recognizing the points of connection between mobile populations and broader society is necessary to access these groups.

Although mobile populations are generally described as hard-to-reach, a number of programmes have successfully worked with mobile populations. For instance one study on the Thai-Cambodia border used respondent driven sampling to contact mobile populations [43]. Many public health projects have recently begun using mobile phone technology to send reminders to health volunteers and workers [24, 51–55]. Another programme in Swaziland successfully used social networks to access mobile contractors [56]. These social networks formed a point of access to the target population and also enabled the researchers to identify other potential sites of transmission at which they could carry out interventions [56]. This experience in Swaziland demonstrates that collaborative community engagement can not only provide access to target populations but may also add important local knowledge to malaria programmes.

The fact that undocumented migrants may not be recognized by the state does not necessarily mean that they are hidden within or marginalized by the communities in which they live. Undocumented mobility, especially near porous borders, is often culturally normalized and social network approaches have also been used to engage with undocumented migrant workers. Singhanetra-Renard suggests that unregistered workers can be reached through cooperation between employers of contract labourer and through recruiting peers of the target group to be health volunteers [19]. The experience of HIV/AIDS programmes has shown that peer educators come forward to support their communities, even at the risk of making themselves vulnerable or disadvantaged [47]. However a collaborative and participative approach is essential for gaining the trust of these peer educators [47–49].

While participatory methods are generally underutilized within malaria prevention, control and elimination activities [57, 58], participatory development approaches have been successfully used in malaria prevention activities in along the Thai-Cambodian border [28]; Vanuatu [57, 59]; Kenya [60]; and Ghana [61]. In a review paper of the use of participatory methods within communicable disease control programmes over sixty years, Atkinson and colleagues demonstrate that community participation has historically played an important role in many successful communicable disease control programmes [58]. Community participation can help to enhance health systems strengthening and can lead to increased resilience against disease by empowering otherwise vulnerable people to prevent disease and access health services [58].

The importance of community participation becomes highly evident as a disease becomes closer to elimination, since public opposition can significantly undermine a disease control programme [58, 59, 62, 63]. This can be seen in the recent challenges to the Global Polio Eradication Initiative, where failed community engagement significantly setback the later phases of the eradication scheme [62, 64]. Aylward and colleagues argue that social and political considerations (including high level political support and community support) have been instrumental to infectious disease control programmes over the past 100 years, but that infectious disease control programmes are yet to fully realize the importance of incorporating socio-political processes into eradication strategies [63], see also [9, 12].

### Imported and border malaria

The third and final theme recurring throughout the literature is imported or border malaria. Once primarily a concern of malaria free countries attempting to prevent reintroduction, imported malaria is now an important concern to many endemic countries, including those working toward elimination [5, 8, 10, 40, 65, 66]. A growing number of researchers and policy makers from eliminating countries are calling for innovative approaches to malaria control that can better address imported malaria, including through a more effective engagement with mobile populations [11, 15, 17, 21, 30, 38, 66, 67]. In addition to these concerns about imported malaria, the paucity of discussion about intra-country mobility and malaria risk suggests that much of the concern about population mobility is focused on imported malaria and border malaria in regions with highly porous borders.

Imported malaria and border malaria is clearly an important issue for some countries. In some regions, such as the GMS, malaria is clustered along forested border areas. In these cases the geography of the border itself increases malaria risk [11, 19]. In addition to geography, border malaria may also be shaped by poverty, which increases exposure to vectors through poor quality clothing, limited access to prevention measures, an increased likelihood to engage in high-risk occupations and difficulties accessing health care [13, 18, 19]. There may also be a lack of health infrastructure in border areas, and in some border regions local communities and migrants alike may not have clear citizenship, which may limit their access to health care and expose both migrants and border communities to risk [32–34]. However while population mobility contributes to imported malaria, these are separate issues that call for different strategies.

Problematically, the literature currently gives a very strong focus on mobile populations as a source of imported malaria with little discussion as to the broader operational challenges of managing imported malaria or to the difficulties that elimination programmes may face working in border areas but see [68]. While interest in regional approaches to malaria elimination is developing [23, 67], it should be remembered that mobile populations are only one factor influencing imported malaria. As recently argued by Williams and colleagues, malaria control programmes ought to take care not to disproportionately attribute blame for imported malaria to mobile populations, but rather should approach mobile populations as stakeholders in malaria elimination [37], see also [23]. Population mobility is a reality of the 21^st^ century and the onus ought to be on malaria control programmes to adapt their technologies to the changing social context of malaria.

## Conclusion

This article has critically reviewed the way that the English language malaria literature perceives the risk surrounding mobile populations and has made recommendations as to how malaria programmes might better conceptualize and respond to population mobility. The article has identified three key themes within the literature: rural economic development and shifting land use; accessing mobile populations; and imported and border malaria. The authors conclude that the risks associated with population mobility are at once overstated and under-examined within the literature. There is currently an excessive focus on mobile populations as a threat to elimination efforts, with inadequate analysis of the circumstances in which population mobility has a significant effect on malaria transmission [8]. The risks assumed to be associated with population mobility are often based on inaccurate assumptions about the behaviours of mobile populations, including overestimations of the speed of mobility, an exaggerated sense of mobile populations as disconnected from communities and an overemphasis on illegality.

The authors recommend that malaria researchers decrease the focus currently given to ‘mobile populations’ as a risk group and instead follow HIV/AIDS programmes in addressing mobility as a system involving multiple demographic groups, localities and intersecting socio-economic processes. Since mobility, by its very nature, creates interconnections between otherwise discrete demographic groups and localities, a strong focus on a particular demographic group will be insufficient to respond to the complexities created by population mobility. The article recommends that concerns about imported malaria may be more productively approached as border health issues, whether intra-country or international borders, since there are likely a range of factors contributing to imported malaria beyond the behaviour of mobile populations *per se*. The authors suggest that collaborative efforts to work with communities along borders and in areas with high population mobility will be more effective than an exclusive focus on the surveillance of mobile populations and specific risk groups. The central shift that is necessary is to move beyond a focus on mobile populations as a demographic group towards approaching and responding to mobility as a multi-faceted system. This conceptual shift will open up space for malaria control programmes to better identify the significance of human population movements on malaria transmission and to engage effectively at different points of these mobility systems.

## Authors’ information

CS is a research assistant for the School of Population Health, University of Queensland and a sessional lecturer in medical anthropology at the University of Queensland. MW is Professor of International and Tropical Health at the University of Queensland, Program Manager of the Australian Initiative for the Control and Elimination of Malaria (AICEM) and Co-Coordinator of the APMEN Secretariat.

## Electronic supplementary material

Additional file 1: **Bibliography accompanying ‘Beyond Mobile Populations’.** (PDF 347 KB)
