# Supplementary material for: Beyond mobile populations: a critical review of the literature on malaria and population mobility and suggestions for future directions
Source: Malar J. 2014 Aug 9;13:307. doi: 10.1186/1475-2875-13-307 (PMC4249613; doi:10.1186/1475-2875-13-307)
Supplement: Supplementary file 1 — Additional file 1: Bibliography accompanying ‘Beyond Mobile Populations’. (PDF 347 KB) [file 12936_2014_3346_MOESM1_ESM.pdf]

**Additional file 1: Bibliography accompanying 'Beyond Mobile Populations'**

Includes material on malaria, HIV and polio published to November 2013. See article for further information on search terms and methods.


Acuin J, Firestone R, Htay TT, Khor GL, Thabrany H, Saphnn V, Wibulpolprasert Sw: **Southeast Asia: An emerging focus for global health.** *Lancet* 2011, **377**:534-535.

Arora NK, Chaturvedi S, Dasgupta R: **Global lessons from India's poliomyelitis elimination campaign**. *Bull World Health Organ* 2010 **88**:232-234.

Asian Development Bank: *Community Action for Preventing HIV/AIDS.* Kingdom of Cambodia, Lao People's Democratic Republic, Socialist Republic of Vietnam: Asian Development Bank, Japanese Fund for Poverty Reduction; 2001.

Asian Development Bank**:** *Development, Poverty and HIV/AIDS: ADB's strategic response to a growing epidemic.* Asian Development Bank; 2005.

http://www.adb.org/documents/development-poverty-and-hiv-aids-adbs-strategic-response-growing-epidemic

Asiimwe C, Gelvin D, Lee E, Amor YB, Quinto E, Katureebe C, Sundaram L, Bell D, Berg M: **Use of an Innovative, Affordable, and Open-Source Short Message Service–Based Tool to Monitor Malaria in Remote Areas of Uganda.** *Am J Trop Med Hyg* 2011, **85**:26-33.

Atkinson J-A, Fitzgerald L, Toaliu H, Taleo G, Tynan A, Whittaker M, Riley I, Vallely A: **Community participation for malaria elimination in Tafea Province, Vanuatu: Part I. Maintaining motivation for prevention practices in the context of disappearing disease.** *Malar J* 2010, **9**:1-16.

Atkinson J-A, Vallely A, Fitzgerald L, Whittaker M, Tanner M: **The architecture and effect of participation: a systematic review of community participation for communicable disease control and elimination. Implications for malaria elimination.** *Malaria J* 2011, **10**:225.

Ayi I, Nonaka D, Adjovu J, Hanafusa S, Jimba M, Bosompem K, Mizoue T, Takeuchi T, Boakye D, Kobayashi J: **School-based participatory health education for malaria control in Ghana: Engaging children as health messengers.** *Malar J* 2010, **9**:1-12.

Aylward B, Hennessey KA, Zagaria N, Olivé J-M, Cochi S: **When is a disease eradicable? 100 years of lessons learned**. *AJPH* 2002, **90**:1515-1520.

Barbieri A, Sawyer I, Soares-Filho B: **Population and Land Use Effects on Malaria Prevalence in the Southern Brazilian Amazon.** *Human Ecology* 2005, **33**:847-874.

Basseri HR, Raeisi A, Holakouie K, Shanadeh K: **Malaria prevention among Afghan refugees in a malarious area, southeastern Iran.** *Bull Soc Pathol Exot* 2010, 103:340-345.

Bhumiratana A, Intarapuk A, Sorosjinda-Nunthawarasilp P, Maneekan P, Koyadun S: **Border Malaria Associated with Multidrug Resistance on Thailand-Myanmar and Thailand-Cambodia Borders: Transmission Dynamic, Vulnerability, and Surveillance.** *BioMed Res Int* 2013:**13**.363417.

Bloland PB, Williams HA. *Malaria Control during Mass Population Movements and Natural Disasters* 2002. Washington, D.C: National Academies Press.

Brentlinger PE **Health, Human Rights and Malaria Control: Historical background and current challenges.** *Health and Human Rights*. 2006. 9.2:10-38.

Bronfman MN, Leyva R, Negroni MJ, Rueda CM: **Mobile populations and HIV/AIDS in Central America and Mexico: research for action**. AIDS 2002, **16**:S42-S49.

Bustos MD, Wongsrichanalai C, Decollette C, Burkholder B. **Monitoring antimalarial drug efficacy in the Greater Mekong Subregion: An overview of in vivo results from 2008 to 2010.** *Southeast Asian J Trop Med and Public Health* 2013.44.1:201-230.

Canavati S, Chea N, Guyant P, Roca-Feltrer A, Yeung S: **Strategy to Address Migrant and Mobile Populations for Malaria Elimination in Cambodia.** Ministry of Health Cambodia, London School of Hygiene and Tropical Medicine, Malaria Consortium; 2013.

Carrara VI, Lwin KM, Phyo AP, Ashley E, Wiladphaingern J, Sriprawat K, Rijken M, Boel M, McGready R, Proux S, et al: **Malaria Burden and Artemisinin Resistance in the Mobile and Migrant Population on the Thai–Myanmar Border, 1999–2011: An Observational Study.** *PLoS Med* 2013, **10**:e1001398.

Chantavanich S: **Mobility and HIV/AIDS in the Greater Mekong Subregion.** Asian Development Bank, United Nations Development Programme, in consortium with World Vision Australia, Macfarlane Burnet Centre for Medical Research; 2000.

Chaveepojnkamjorn W, Pichainarong N: **Malaria infection among the migrant population along the Thai-Myanmar border area.** *Southeast Asian J Trop Med Public Health* 2004, **35**:48-52.

Chaveepojnkamjorn W, Pichainarong N: **Behavioral Factors and Malaria Infection among the Migrant Population, Chiang Rai Province.** *J Med Assoc Thai* 2005, **99**:1293-1301.

Cochi SL, Linkins RW: **The Final Phase of Polio Eradication: New Vaccines and Complex Choices.** *J Infect Dis* 2012, **205**:169-171.

Coker RJ, Hunter BM, Rudge JW, Liverani M, Hanvoravongchai P: **Health in Southeast Asia 3: Emerging infectious diseases in southeast Asia: Regional challenges to control.** *Lancet* 2011, **377**:599-609.

Cotter C, Sturrock HJW, Hsiang MS, Liu J, Phillips AA, Hwang J, Smith Gueye C, Fullman N, Gosling RD, Feachem RGJ: **The changing epidemiology of malaria elimination: new strategies for new challenges.** *Lancet* 2013, **382**:900-911

Cui L, Yan G, Sattabongkot J, Cao Y, Chen B, Chen X, Fan Q, Fang Q, Jongwutiwes S, Parker D, et al: **Malaria in the Greater Mekong Subregion: Heterogeneity and Complexity.** *Acta Trop* 2012, **121**:227-239.

Darby E, Parnell B, Minn K: **Toolkit for HIV prevention among mobile populations in the Greater Mekong Subregion.** Asian Development Bank, UNDP; 2002.

Delacollette C, D'Souza, C, Christophel E, Thimasarn K, Abdur, R, Bell D, Dai TC, Gopinath D, Lu S, Mendoza R, Ortega L, Rastogi R, Tantinimitkul C, Ehrenberg J. **Malaria Trends and Challenges in the Greater Mekong Subregion.** *Southeast Asian J Trop Med and Public Health.* 2009.40.4.July:674-691.

Deressa W, Ali A, Berhane Y: **Review of the interplay between population dynamics and malaria transmission in Ethiopia.** *EJHD* 2006, **20**:137-134.

Durnez L, Mao S, Denis L, Roelants P, Sochantha T, Coosemans M: **Outdoor malaria transmission in forested villages of Cambodia.** *Malar J* 2013, **12**:329.

Dysoley L, Kaneko A, Eto H, Mita T, Socheat D, Börkman A, Kobayakawa T: **Changing patterns of forest malaria among the mobile adult male population in Chumkiri District, Cambodia.** *Acta Trop* 2008, **106**:207-212.

Feachem RGA, Phillips AA, Hwang J, Cotter C, Wielgosz B, Greenwood BM, Sabot O, Rodriguez MH, Abeyasinghe RR, Ghebreyesus TA, Snow RW. **Shrinking the Malaria Map: Progress and Prospects.** *The Lancet* 2010.376.9752:1566-1578.

Gilbert L, Walker L: **Treading the path of least resistance: HIV/AIDS and social inequalities - a South African case study**. *Soc Sci Med* 2002, **54**:1093-1110.

Gosling RD, Whittaker M, Gueye CS, Fullman N, Baquilod M, Kusriastuti R, Feachem RG: **Malaria elimination gaining ground in the Asia Pacific.** *Malar J* 2012, **11**:346.

Gushulak BD, MacPherson DW: **Globalization of Infectious Diseases: The Impact of Migration.** *Clin Infect Dis* 2004, **38**:1742-1748.

Gushulak BD, MacPherson DW: **The basic principles of migration health: Population mobility and gaps in disease prevalence.** *Emerg Themes Epidemiol* 2006, **3**:10.1186/1742-7622-3-3.

Haour-Knipe M, Fleury F, Dubois-Arber F: **HIV/AIDS prevention for migrants and ethnic minorities: Three phases of evaluation.** *Soc Sci Med* 1999, **49**:1357-1372.

Hay SI, Guerra CA, Tatem AJ, Noor AM, Snow RW: **The global distribution and population at risk of malaria: past, present, and future.** *Lancet Infect Dis* 2004, **4**:327-336.

Hewitt S, Delacollette C, Chavez I: **Malaria Situation in the Greater Mekong Subregion.** *Southeast Asian J Trop Med Public Health* 2013, **44**:46-72.

Hugo G: *Indonesia. Internal and International Population Mobility: Implications for the spread of HIV/AIDS.* UNDP Southeast Asia HIV and Development Office; ILO, Indonesia; UNAIDS, Indonesia; 2001.

International Organization for Migration*: Compendium of Migration and HIV and Aids Interventions.* (Compiled by Jose Michael, Malathy EbPN eds.): International Organization for Migration, with UNDP and UNAIDS. Geneva, Switzerland: 2009.

Jitthai N: **Migration and Malaria.** *Southeast Asian J Trop Med Public Health* 2013, **44**:166-200.

Khamsiriwatchara A, Wangroongsarb P, Thwing J, Eliades J, Satimai W, Delacollette C, Kaewkungwal J: **Respondent-driven sampling on the Thailand-Cambodia border. 1. Can malaria cases be contained in mobile migrant workers?** *Malar J* 2011, **10**:120.

Kitvatanachai S, Janyapoon K, Rhongbutsri P, Thap L: **A survey on malaria in mobile Cambodians in Aranyaprathet, Sa Kaeo Province, Thailand.** 2003 *Southeast Asian J Trop Med Public Health* 2003, **34**:48-53.

Koita K, Novotny J, Kunene S, Zulu Z, Ntshalintshali N, Gandhi M, Gosling R. **Targeting imported malaria through social networks: a potential strategy for malaria elimination in Swaziland.** *Malaria J* 2013.12:219.

MacPherson DW, Gushulak BD: **Human Mobility and Population Health: New Approaches in a Globalizing World.** *Perspect Biol Med* 2001, **44**:390-401.

MacPherson DW, Gushulak BD, Macdonald L: **Health and foreign policy: influences of migration and population mobility**. *Bull World Health Organ* 2007, **85**:200-206.

Manimunda SP, Sugunan AP, Sha WA, Singh SS, Shriram AN, Vijayachari P: **Tsunami, post-tsunami malaria situation in Nancowry group of islands, Nicobar district, Andaman and Nicobar Islands.** *Indian J Med Res* 2011, **133**:76-82.

Martens P, Hall L: **Malaria on the Move: Human population movement and malaria transmission.** *Emerg Infect Dis* 2000, **6**:103-109.

McElroy B, Wiseman V, Matovu F, Mwengee W: **Malaria prevention in north-eastern Tanzania: Patterns of expenditure and determinants of demand at the household level.** *Malar J* 2009, **8**:1-10.

McLeod M: **"We Cubans are obliged like cats to have a clean face": Malaria, quarantine and race in neocolonial Cuba, 1898-1940.** *The Americas* 2010, **67**:57-81.

McMichael AJ, Beaglehole R: **The changing global context of public health**. *Lancet* 2000, **356**:495-499.

Meankaew P, Kaewkungwal J, Khamsiriwatchara A, Khunthong P, Singhasivanon P, Satimai W: **Application of mobile-technology for disease and treatment monitoring of malaria in the "Better Border Healthcare Programme".** *Malar J* 2010, **9**:237.

International Organization of Migration: **Global Report on Population Mobility and Malaria: Moving towards elimination with migration in mind.** International Organization for Migration; 2013.

Moonen B, Cohen JM, Snow RW, Slutsker L, Drakeley C, Smith DL, Abeyasinghe RR, Rodriguez MH, Maharaj R, Tanner M, Targett G: **Malaria Elimination 3: Operational strategies to achieve and maintain malaria elimination.** *Lancet* 2010, **376**:1592-1603.

Moonena B, Cohen JM: **Text messaging to improve adherence to malaria guidelines**. *Lancet* 2011, **378**:750-752.

Moore S, Min X, Hill N, Jones C, Zaixing Z, Cameron M: **Border malaria in China: knowledge and use of personal protection by minority populations and implications for malaria control: a questionnaire-based survey.** *BMC Public Health* 2008, **8**:344-352.

Ngom R, Siegmund A: **Urban malaria in Africa: an environmental and socio-economic modelling approach for Yaoundé, Cameroon.** *Nat Hazards* 2010, **55**:599-619.

Nishtar S. **Pakistan, politics and polio**. *Bulletin of the World Health Organization* 2010.88:159-160.

Obrist B, Mayumana I, Kessy F: **Livelihood, malaria and resilience.** *PIDS* 2010, **10**:325-343.

World Health Organization: **World Malaria Report 2011**. Geneva, Switzerland: World Health Organization; 2011.

World Health Organization: **Session 6. Background Paper. Biregional Meeting on Healthy Borders in the Greater Mekong Subregion 5-7 August**. Bangkok, Thailand: World Health Organization; 2013.

O'Shannassy T: **Greater Mekong Subregion (GMS): Context**. *Southeast Asian J Trop Med Public Health* 2013, **44**:1-45.

Opiyo P, Mukabana W, Kiche I, Mathenge E, Killeen G, Fillinger U. **An exploratory study of community factors relevant for participatory malaria control on Rusinga Island, Western Kenya** *Malaria Journal* 2007.6.1:1-20.

Osorio L, Todd J, Bradley DJ: **Travel Histories as Risk Factors in the Analysis of Urban Malaria in Colombia.** *Am J Trop Med Hyg* 2004, **71**:380-386.

Pattanasin S, Satitvipawee P, Wongklang W, Viwatwongkasem C, Bhumiratana A, Soontornpipit P, Jareinpituk S: **Risk Factors for Malaria Infection Among Rubber Tappers living in a Malaria Control Program area in Southern Thailand**. *Southeast Asian J Trop Med Public Health* 2012, **43**:1313-1325.

Pichainarong N, Chaveepojnkamjorn W: **Malaria infection and life-style factors among hilltribes along the Thai-Myanmar border area, northern Thailand**. *Southeast Asian J Trop Med Public Health* 2004, **35**:834-839.

Pindolia DK, Garcia AJ, Huang Z, Smith DL, Alegana VA, Noor AM, Snow RW, Tatem AJ: **The demographics of human and malaria movement and migration patterns in East Africa.** *Mal J* 2013, **12**:397.

Pindolia DK, Garcia AJ, Wesolowski A, Smith DL, Buckee CO, Noor AM, Snow RW, Tatem AJ: **Human movement data for malaria control and elimination strategic planning.** *Mal J* 2012, **11**:205.

Prothero R: **Population Movements and Tropical Health.** *CGHH* 2002, **3**:20-32.

Prothero RM: **Migration and malaria risk.** *Health Risk Soc* 2001, **3**:19-38.

Prothero RM: **Malaria and the Importance of People**. *DIP* 2001, **11**:86-91.

Puri M, Cleland J: **Sexual behavior and perceived risk of HIV/AIDS among young migrant factory workers in Nepal.** *J Adolesc Health* 2006, **38**:237-246.

Renne EP: *The Politics of Polio in Northern Nigeria.* Bloomington, Indiana: Indiana University Press; 2010.

Richard G: **Malaria control in Nicaragua: Social and political influences on disease transmission and control activities.** *Lancet* 1999, **354**:414-418.

Rodríguez-Morales AJ, Delgado L, Martínez N, Franco-Paredes C: **Impact of Imported Malaria on the Burden of Disease in Northeastern Venezuela.** *J Travel Med* 2006, **13**:15-20.

Sachs J, Malaney P: **The economic and social burden of malaria.** *Nature* 2002, **415**:680-685.

Schlagenhauf P, Hommel M: **Travellers' malaria - 'one shoe does not fit all'.** *Mal J* 2011, **10**:129.

Sevilla-Casas E: **Human mobility and malaria risk in the Naya River Basin of Colombia.** *Soc Sci Med* 1993, **37**:1155-1167.

Singhanetra-Renard A: **Malaria and mobility in Thailand**. *Soc Sci Med* 1993, **37**:1147-1154.

Singhasivanon P: **Mekong malaria. Malaria, multi-drug resistance and economic development in the Greater Mekong Subregion of Southeast Asia.** *Southeast Asian J Trop Med Public Health* 1999, **30,** Suppl:i-iv.

Skeldon R: *Population Mobility and HIV Vulnerability in Southeast Asia: An assessment and analysis.* UNDP Southeast Asia HIV and Development. Bangkok, Thailand; 2000.

Smith DL, Cohen JM, Chiyaka C, Johnston G, Gething PW, Gosling R, Buckee CO, Laxminarayan R, Hay SI, Tatem AJ: **A sticky situation: the unexpected stability of malaria elimination.** *Phil Trans R Soc B* 2013, 368:20120145.

Smith Gueye C, Teng A, Kinyua K, Wafula F, Gosling R, McCoy D: **Parasites and Vectors Carry no Passport: How to fund cross-border and regional efforts to achieve malaria elimination.** *Mal J* 2012, 11.

Sturrock H, Roberts K, Ohrt C, Wegbreit J, Gosling, R. *Background Paper. Effective Responses to Malaria Importation.* 2014. Global Health Group.

Socheat D DM, Fandeur T, Zhang Z, Yang H, Xu J, Zhou X, Phompida S, Phetsouvanh R, Lwin S, Lin K, Win T, Than SW, Htut Y, Prajakwong S, Rojanawatsirivet C, Tipmontree R, Vijaykadga S, Konchom S, Cong le D, Thien NT, Thuan le K, Ringwald P, Schapira A, Christophel E, Palmer K, Arbani PR, Prasittisuk C, Rastogi R, Monti F, Urbani C, Tsuyuoka R, Hoyer S, Otega L, Thimasarn K, Songcharoen S, Meert JP, Gay F, Crissman L, Cho-Min-Naing, Chansuda W, Darasri D, Indaratna K, Singhasivanon P, Chuprapawan S, Looareesuwan S, Supavej S, Kidson C, Baimai V, Yimsamran S, Buchachart K.: **Mekong malaria. II. Update of malaria, multi-drug resistance and economic development in the Mekong region of Southeast Asia.** *Southeast Asian J Trop Med Public Health* 2003, **34**, Suppl:1-102.

Spiegel PB: **HIV/AIDS among Conflict-affected and Displaced Populations: Dispelling Myths and Taking Action.** *Disasters* 2004, **28**:322-339.

Stoddard ST, Morrison AC, Vazquez-Prokopec GM, Soldan VP, Kochel TJ, Kitron U, Elder JP, Scott TW: **The Role of Human Movement in the Transmission of Vector-Borne Pathogens.** *PLoS Negl Trop Dis* 2009, **3**:e481.

Tatem AJ, Qiu Y, Smith DL, Sabot O, Ali AS, Moonen B: **The use of mobile phone data for the estimation of the travel patterns and imported *Plasmodium falciparum* rates among Zanzibar residents.** *Mal J* 2009, **8**:287.

Tatem AJ, Smith DL: **International population movements and regional Plasmodium falciparum malaria elimination strategies.** *PNAS* 2010, **107**:12222–12227.

Terry M: **Text Messaging in Healthcare: The Elephant Knocking at the Door.** *Telemed J E Health* 2008, **14**:520-524.

Tipmontree R, Fungladda W, Kaewkungwal J, Tempongko MA, FP S: **Migrants and malaria risk factors: A study of the Thai-Myanmar border.** *Southeast Asian J Trop Med Public Health* 2009, **40**:1148-1157.

Tynan A, Atkinson J-A, Toaliu H, Taleo G, Fitzgerald L, Whittaker M, Riley I, Schubert M, Vallely A: **Community participation for malaria elimination in Tafea province, Vanuatu: Part ii. Social and cultural aspects of treatment-seeking behaviour**. *Mal J* 2011, **10**:1-12.

Wangroongsarb P, Satimai W, Khamsiriwatchara A, Thwing J, Eliades JM, Kaewkungwal J, Delacollette C: **Respondent-driven sampling on the Thailand-Cambodia border. II. Knowledge, perception, practice and treatment-seeking behaviour of migrants in malaria endemic zones.** *Mal J* 2011, **10**:117.

Wesolowski A, Eagle N, Tatem AJ, Smith DL, Noor AM, Snow RW, Buckee CO: **Quantifying the Impact of Human Mobility on Malaria.** *Science* 2012, **338**:267-270.

Wickramage K, Premaratne RG, Peiris SL, Mosca D: **High attack rate for malaria through irregular migration routes to a country on verge of elimination.** *Mal J* 2013, **12**:276.

Williams HA, Hering H, Spiegel PB: **Discourse on malaria elimination: Where do forcibly displaced persons fit in these discussions?** *Mal J* 2013, **12**:121.

World Health Organization: **World Malaria Report 2011**. Geneva, Switzerland: World Health Organization; 2011.

World Health Organization: **Community-based reduction of malaria transmission: Informal consultation organized by the WHO Global Malaria Programme in collaboration with Malaria Eradication Research Agenda (malERA) initiative.** World Health Organization; 2012.

World Health Organization: Session 6. *Background Paper. Biregional Meeting on Healthy Borders in the Greater Mekong Subregion* 5-7 August. Bangkok, Thailand: World Health Organization; 2013.

http://www.searo.who.int/thailand/news/healthbordermeeting/en/index.html

Yangzom T, Smith Gueye C, Namgay R, Galappaththy GN, Thimasarn K, Gosling R, Murugasampillay S, Dev V: **Malaria control in Bhutan: case study of a country embarking on elimination.** *Mal J* 2012, 11:9.

Yin J-h, Yang M-n, Zhou S-s, Wang Y, Jun Feng, Xia Z-g: **Changing Malaria Transmission and Implications in China towards National Malaria Elimination Programme between 2010 and 2012.** *PLOS ONE* 2013, **8**:e74228.

Zurovac D, Sudoi RK, Akhwale WS, Ndiritu M, Hamer DH, Rowe AK, Snow RW: **The effect of mobile phone text-message reminders on Kenyan health workers' adherence to malaria treatment guidelines: A cluster randomised trial.** *Lancet* 2011, **378**:795-803.

Zurovac D, Talisuna AO, Snow RW: **Mobile Phone Text Messaging: Tool for Malaria Control in Africa.** *PLoS Med* 2012, **9**:e1001176.
